# The association between periodontal microbial biomarkers and primary therapy outcome

**DOI:** 10.1007/s00784-024-05904-y

**Published:** 2024-09-13

**Authors:** Nils Werner, Iris Frasheri, Katrin Heck, Christina Ern, Richard Heym, Caspar Victor Bumm, Matthias Folwaczny

**Affiliations:** 1 Department of Conservative Dentistry and Periodontology, University Hospital, LMU Goethestr. 70, 80336 Munich, Germany; 2 Private practice, Munich, Germany

**Keywords:** Periodontal therapy, Periodontitis, Biomarker, Inflammation

## Abstract

**Objective:**

This study aims to analyse the association between the baseline microbial load of selected periodontopathogenic bacteria collected from gingival crevicular fluid (GCF) and the primary outcome of steps I and II therapy.

**Materials and methods:**

222 patients with stage III periodontitis were included into this retrospective analysis that received steps 1 and 2 periodontal therapy without adjunctive systemic antibiotics. Baseline GCF samples were quantitatively analysed using ELISA-based kits for levels of periodontopathogens (*Porphyromonas gingivalis* (*Pg*), *Aggregatibacter actinomycetemcomitans* (*Aa*), *Prevotella intermedia* (*Pi*), *Fusobacterium nucleatum* (*Fn*), *Treponema denticola* (*Td*), and *Tannerella forsythia* (*Tf*)) and associated with the primary therapy outcome using a “treat-to-target” therapy endpoint (TE) defined as ≤ 4 sites with PD ≥ 5 mm six months after therapy.

**Results:**

38.2% of the patients achieved TE. Patients failing to achieve TE revealed significantly increased levels of *Pg*, *Fn*, and *Tf* at baseline (*Pg*: *p* = 0.010, *Fn*: *p* = 0.008 *Tf*: *p* = 0.004). Multivariate binary logistic regression adjusted for sex, mean probing depth, diabetes, and current smoking status showed an independent relationship between *Tf* and the TE (aOR 2.570, *p* = 0.023).

**Conclusion:**

Increased microbial load is associated with decreased responsiveness to therapy. The findings suggest that specifically baseline *Tf* levels are associated with poorer treatment outcomes and might improve the accuracy of periodontal diagnosis.

**Clinical relevance:**

The findings of this study support the concept of a critical biomass that is sufficient to induce and maintain an immune response within the periodontal pocket, which ultimately leads to irreversible tissue destruction. However, calculating this level in advance may serve as an early indicator for intervention.

**Key finding:**

Baseline *Tannerella forsythia* levels are associated with poorer treatment outcome.

## Introduction

The onset of periodontitis centrally involves a dysbiotic shift in the subgingival microbiota, along with an exaggerated immune-inflammatory infiltration within the periodontium that is largely host-specific [1, 2]. Steps I and II are the initial cause-related phases of therapy, which has proven to be effective in reducing inflammation and should be performed regardless of the stage or grade of the disease as first-line therapy [3–7]. It is commonly accepted that the individual success of this therapeutic intervention shows is poorly predictable and shows great varity among patients [8–10]. For this reason, the current classification of periodontal and peri-implant diseases and conditions introduced a grading system, which aims to allow for improved estimation of the response to standard therapy [11]. This graduation system is primarily based on the assessment of previous periodontal destruction, with particular emphasis on the past five years for each patient, and on the presence of major risk factors like diabetes and smoking [12, 13]. Tonetti et al. also presented the possibility of using additional parameters, i.e. the clinical phenotype or systemic biomarkers for periodontal grading [11]. In this context, the overwhelming majority of biomarkers comprise cytokines and chemokines, particularly expressed by the diseased periodontal tissue in response to a dysbiotic subgingival microbiome [1, 2, 14, 15]. Moreover, the individual pattern of the subgingival microbiome itself might provide insight into the disease status of the periodontal pocket and as stated by Manoil et al. a potentially dysbiotic onset could possible detected [16, 17].

The role of subgingival microbiota in periodontitis is complex. However, Curtis et al. proposed three essential hypotheses that are based on a broad consensus [18]. Firstly, bacteria are required in the development of periodontitis [18, 19]. Secondly, changes in the microbial community of the subgingival biofilm and bacterial load are associated with periodontal destruction [18, 20]. Thirdly, an excessive inflammatory host response is responsible for periodontal destruction [18, 21]. Accordingly, steps I and II therapy is centrally directed towards the elimination of both, inflammation and bacterial colonization [3–7]. Microbial pathogens have also been used in the past to determine the efficacy of adjunctive systemic antibiotics during step II therapy, however, only the presence or absence of distinct bacteria has been considered [17, 22]. Yet, due to the high complexity of the subgingival microbiome this approach proved to be too simplified [18, 22, 23]. Nevertheless, Belibasakis et al. have highlighted the potential of quantifying periodontopathogenic bacteria to enhance periodontal diagnosis and inform treatment planning [23].

Due to partially conflicting data and insufficient evidence, this study aimed to associate the baseline levels of the periodontopathogenic bacteria with the individual response to steps I and II therapy using a predefined endpoint variable.

## Methods

### Study design and source of data

The clinical trial was approved by the Ethics Committee of the Medical Faculty of the Ludwig Maximilian University, Munich, Germany (No. 025 − 11) and conducted following the principles of good clinical practice and the Declaration of Helsinki. Reporting of this study follows the STROBE guidelines [24].

This retrospective analysis of a prospective study observed 222 patients, who were enrolled into steps I and II therapy for treatment of periodontitis in the undergraduate course at the Department of Conservative Dentistry and Periodontology, University Hospital, LMU Munich between February 2011 and March 2016 [7].

All study subjects received steps I and II therapy treatment upon diagnosis of periodontitis for the first time or of recurrent disease following previous periodontal treatment. Patients had to meet the following inclusion criteria: (1) age ≥ 18 years, (2) stage III periodontitis according to the current classification [11], (3) periodontal chart with documentation of probing pocket depth (PPD) and bleeding on probing (BOP) at six sites/tooth before steps I and II therapy, (4) periodontal chart with documentation of probing pocket depths and bleeding on probing at six sites/tooth at re-evaluation (REV), (5) laboratory analysis of baseline GCF samples considering six periodontopathogenic bacteria (*Porphyromonas gingivalis (Pg), Aggregatibacter actinomycetemcomitans (Aa), Prevotella intermedia (Pi), Fusobacterium nucleatum (Fn), Treponema denticola (Td)*, and *Tannerella forsythia (Tf)*). The exclusion criteria were as follows: (1) pregnancy at baseline, (2) previous periodontal treatment < 2 years prior to enrolment into the study, (3) current enrolment into supportive periodontal therapy (SPT), (4) indication for systemic antibiotics as an adjunctive to steps I and II therapy.

### Periodontal treatment

Periodontal treatment was described in detail by Werner et al. before [7]. In brief: Patients received comprehensive information regarding the aetiology, pathogenesis, risk factors, and treatment of periodontitis. Furthermore, as part of step I, oral hygiene instructions and professional mechanical plaque removal were performed. Subgingival debridement was carried out under local anaesthesia for all teeth with PPD > 3 mm, using SonicFLEX (KaVo Dental, Biberach, Germany) together with a standardized set of Gracey curettes (SG5/6, SG7/8, SG 13/14, SG15/16 (Hu-Friedy, Chicago, USA)), without restrictions in time [7, 8].

### Analysis of pathogenic bacteria

The procedure for collecting GCF and pathogen analysis has been previously described in detail [25]. In brief, samples were obtained from the deepest periodontal pockets of each quadrant using sterile paper points and pooled for further analysis. The bacterial DNA isolation from the samples was conducted using the MagNA Pure DNA Isolation Kit III (Roche Diagnostics, Mannheim, Germany), following the instructions provided by the manufacturer. Amplification of the DNA was performed using the Parident-kit (AMPLEX Diagnostics, Gars am Inn, Germany) according to the protocol as described by Frasheri et al. [25]. For each group of bacteria, 5 µl of the DNA sample were mixed with 45 µl of the corresponding master mix. This was followed by a hybridization-based detection or probe hybridization assay. The stranded amplicons of each sample were then transferred into colour-coded wells, specific for the six tested pathogens. After incubation with hybridisation buffer, peroxidase conjugate was added to the reaction. In a further step, a chromogenic substrate, 3,3’,5,5’-Tetramethylbenzidine was used to identify the peroxidase conjugate bound to the biomarkers. The change in optical density (OD) of the samples was measured with the spectrophotometer Varioskan 3.00.7 (Thermo Fisher Scientific, Waltham, MA, USA) at 450 nm and 620 nm.

### Clinical parameters and outcome variables

Periodontal examination was conducted prior to steps I and II therapy (baseline, T0) and after 6 months (REV, T1) [7]. PPD was measured to the nearest millimetre using a PCP-12 periodontal probe with a trained probing force of 0.2–0.3 N [26]. BOP was determined approximately 30 s after probing [27]. Mobility was assessed according to Miller [28]. Furcation involvement (FI) was measured with a 2 N-Nabers probe and graded as described by Hamp et al. [29]. Periodontal classification was determined using the 2018 classification [11]. At the site level proportions of periodontal pockets (PPD%) were calculated at baseline and re-evaluation using the parameter pocket closure (PC) defined per site, as a PPD of 4 mm in the absence of BOP or ≤ 3 mm, as stated by the current classification [30]. Furthermore, the differentiated BOP thresholds (< 10%, 10–20% and > 20%), were set, according to Feres et al. [31]. Smoking status is defined as current smoking or non-smoking. As the primary outcome variable the therapy endpoint (TE) was defined as suggested by Feres et al. as ≤ 4 sites with PPD ≥ 5 mm [31].

### Sample size

Sample size calculation was done with G-Power calculator (version 3.1) based on the data as previously reported by Byrne et al. assuming an effect size d of 0.9 based on the relative bacterial mass as found for *Pg* at baseline at sites without treatment success and control sites [32]. Accordingly, a minimum sample size of 54 has to be considered to reach a power of 0.9.

### Source of bias

Periodontal diagnosis and treatment were carried out in the undergraduate programme. To ensure sufficient quality of therapy, undergraduate students underwent extensive training in advance [33, 34]. In addition, all steps of therapy and diagnosis were supervised by two experienced dentists (CE and RH) calibrated for periodontal probing in advance [33, 34].

### Statistical analysis

Numerical data are expressed as mean (± SD), categorical variables are presented as absolute and relative frequencies (percentages). Non-normally distributed variables are presented as median and interquartile range [q1;q3]. The normality of data was tested using the Shapiro-Wilk test. For univariate analysis differences between patients were compared using Student’s t-test for continuous variables, Mann-Whitney U test for ordinal and skewed variables, and Chi-squared test for categorical variables. Logistic regression models were used to identify potential confounders of the TE. For multivariate analysis, a binary logistic regression model was employed including all pathogenic bacteria identified in univariate analysis and possible confounders. Results are shown as adjusted odds ratios (aOR) per 1-unit change of PC with corresponding 95% CIs. Using the dichotomous variable TE, ROC-analysis was done and the area under the curve (AUROC) was computed. For the delineation of threshold values, the Youden index has been calculated. The two-sided significance level was set at α = 0.05 for all tests. All analyses were performed using SPSS (Version 29.0, IBM, Armonk, USA).

## Results

### Patient characteristics

Seven hundred fifty-nine patients received steps I and II therapy between February 2011 and March 2016. The final analysis included 222 patients showing a mean age of 59.5 (± 11.4) years. The male-to-female ratio was 54.5/45.5%, 24.3% of study subjects were current smokers, and 9.0% had been diagnosed with diabetes mellitus (Table 1). Patients presented with a mean PPD of 2.78 ± 0.55 mm and with a total of 21.3 ± 15.4% periodontal pockets at baseline. Of the 206 patients eligible for grading 7 (3.4%) could be classified as grade A, 137 (66.5%) as grade B and 62 (30.1%) as grade C.

**Table 1:** Baseline characteristics

Variable	Total (*n* = 222)
Age, y	59.5 ± 11.4
Female, n (%)	101 (45.5)
Male, n (%)	121 (54.5)
Non-diabetic, n (%)	202 (91.0)
Diabetic, n (%)	20 (9.0)
Non-smokers, n (%)	168 (75.7)
Smokers, n (%)	54 (24.3)
Number of teeth per patient, n	22.6 ± 11.4
Periodontal grade, n (%)	
Grade A	7 (3.4)
Grade B	137 (66.5)
Grade C	62 (30.1)
Mean probing pocket depth baseline, mm	2.78 ± 0.55
PPD%Base, %	21.3 ± 15.4
*Aa*, OD [q1;q3]	0.05 [0.03;0.11]
*Pg*, OD [q1;q3]	3.02 [0.26;4.04]
*Fn* OD [q1;q3]	1.03 [0.37;1.89]
*Pi*, OD [q1;q3]	0.23 [0.09;0.73]
*Tf*, OD [q1;q3]	0.24 [0.08;0.56]
*Td*, OD [q1;q3]	0.64 [0.09;1.49]

*Aa, Aggregatibacter actinomycetemcomitans*; BOP, bleeding on probing;* Pg, Porphyromonas gingivalis; Pi, Prevotella intermedia; Fn, Fusobacterium nucleatum;* OD, optical density; PPD%Base, sites with periodontal pockets in %; *Td, Treponema denticola; Tf, Tannerella forsythia*

### Periodontal infection and therapy endpoints

After steps I and II therapy patients presented with significantly lower proportion of sites with periodontal pockets compared to baseline (21.3 ± 15.4% vs. 14.6 ± 12.4%, *p* < 0.001) (Table 2). 61.3% of patients failed to reach the TE at re-evaluation. Among these, 26.5% were current smokers and 9.6% had diabetes (Table 2). At REV, 16.2% of all patients showed a BOP of < 10%, 22.1% a BOP of 10–20% and in 61.7% presented with a BOP > 20% of all sites.

**Table 2:** Periodontal status at re-evaluation

Variable	Total (*n* = 222)
PPD%REV, %	14.6 ± 12.4
BOP, %	27.4 ± 17.2
Patients without residual pockets, n(%)	2 (0.9)
Patients not reached therapy endpoint at re-evaluation, n (%)	136 (61.3)
-with diabetes, n (%)	13 (9.6)
-smoking, n (%)	36 (26.5)
% BOP endpoint	
BOP ≤ 10%, n (%)	36 (16.2)
BOP > 10%, n (%)	49 (22.1)
BOP > 20%, n (%)	137 (61.7)

BOP, bleeding on probing; PPD%REV, sites with periodontal pockets in % at re-evaluation

**Fig. 1:**
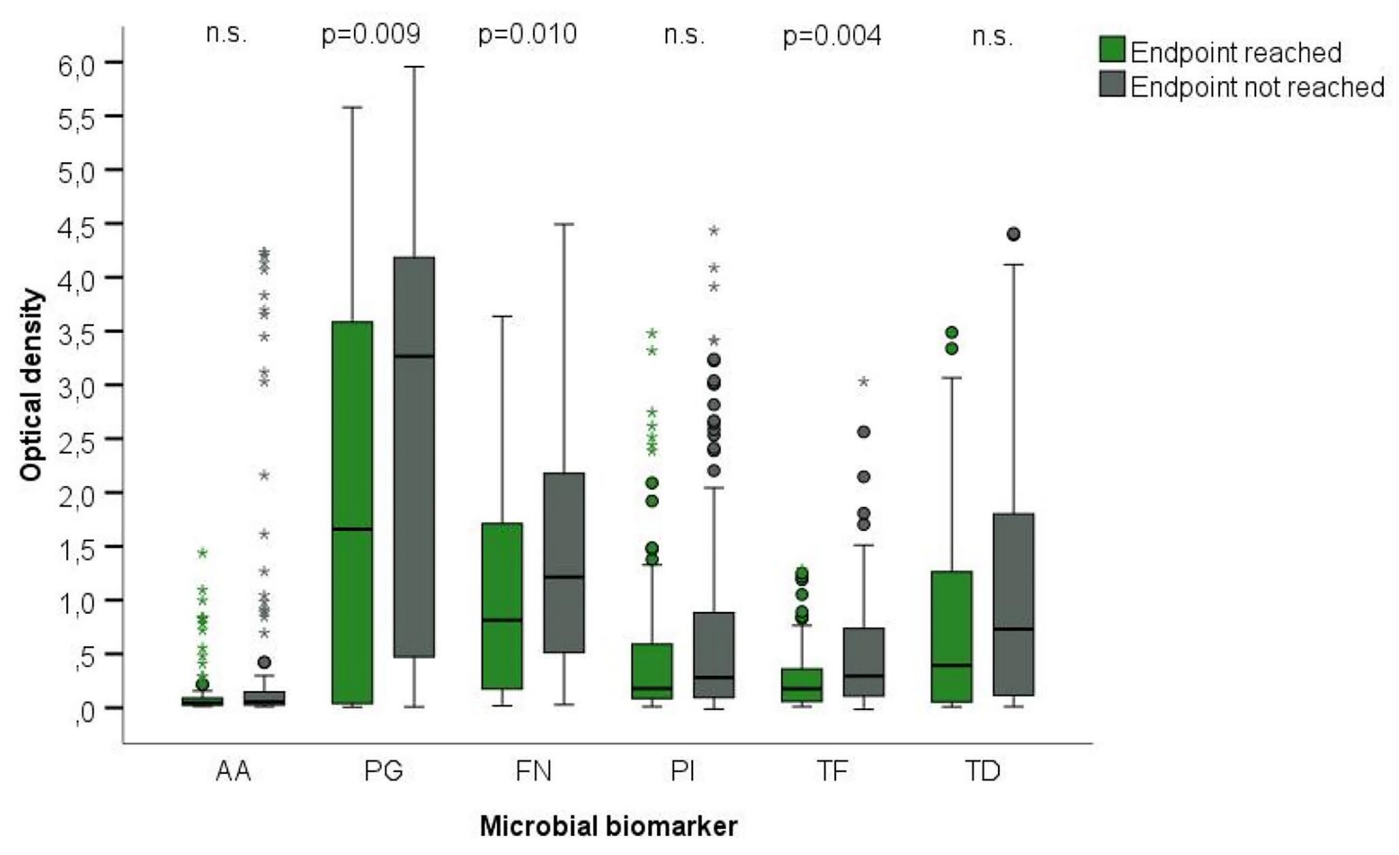
Box-Whiskers-Plot of microbial biomarkers (*Porphyromonas gingivalis* (*Pg*), *Aggregatibacter actinomycetemcomitans* (*Aa*), *Prevotella intermedia* (*Pi*), *Fusobacterium nucleatum* (*Fn*), *Treponema denticola* (*Td*), and *Tannerella forsythia* (*Tf*)) measured in ∆ absorbance 440–620 nm. Biomarkers are collected from gingival crevicular fluid (GCF). The box extends from the lower to the upper quartile and the two whiskers indicate the minimum and maximum. The median is drawn as a horizontal line inside the box. If the whiskers are longer than 1.5 times the box, all values that exceed this are labelled as outliers (stars and dots)

In patients who did not reach TE, a significantly higher OD of *Pg*, *Fn* and *Tf* was detected at baseline (*Pg*: *p* = 0.010, *Fn*: *p* = 0.008 *Tf*: *p* = 0.004). No significant differences in OD were detected between the various levels of BOP for any of the tested bacteria (Fig. 1; Table 3).

**Table 3:** Optical density of different pathogens across patients’ BOP level

Factor	*n*	All	BOP < 10%	BOP 10–20%	BOP > 20%	*p*-value
*Aa*	222	0.05 [0.03;0.11]	0.05 [0.03;0.09]	0.04 [0.02;0.10]	0.05 [0.03;0.15]	0.539
*Pg*	222	3.02 [0.26;4.04]	3.05 [0.12;4.12]	2.29 [0.07;4.01]	3.08 [0.27;4.12]	0.874
*Fn*	222	1.03 [0.37;1.89]	1.22 [0.42;2.56]	0.78 [0.23;1.69]	1.04 [0.47;1.97]	0.248
*Pi*	222	0.23 [0.09;0.73]	0.33 [0.13;0.88]	0.21 [0.08;0.52]	0.21 [0.09;0.84]	0.287
*Tf*	222	0.24 [0.08;0.56]	0.25 [0.08;0.42]	0.21 [0.07;0.54]	0.25 [0.08;0.65]	0.742
*Td*	222	0.64 [0.09;1.49]	0.69 [0.07;1.82]	0.44 [0.05;1.32]	0.69 [0.11;1.58]	0.529

Data are present as median [q1;q3]

*Aa*, *Aggregatibacter actinomycetemcomitans*; BOP, bleeding on probing; *Pg*, *Porphyromonas gingivalis*; *Pi*, *Prevotella intermedia*; *Fn*, *Fusobacterium nucleatum*; *Td*, *Treponema denticola*; *Tf*, *Tannerella forsythia*

Logistic regression further corroborated these findings. Using a univariate logistic regression model, a potential association between therapeutic outcome and bacterial infection was found for *Aa* (OR 1.831, *p* = 0.031), *Pg* (OR 1.182, *p* = 0.028), *Fn* (OR 1.021, *p* = 0.034) and *Tf* (OR 1.130, *p* = 0.003). Considering sex, diabetes, mean PPD and current smoking status as confounders a multivariate analysis revealed that higher amounts of *Tf* (aOR 2.570, *p* = 0.023) were significantly and independently associated with failing TE at REV (Table 4).

**Table 4:** Binary logistic regression model – dependent variable TE at re-evaluation

Variabels	Univariable logistic regression		Multivariate logistic regression	
Independent	OR (95% CI)	p-value	aOR (95% CI) adjusted for sex, mean PPD, diabetes and smoking	p-value
*Aa*	1.801(1.056;3.069)	**0.031**	1.695(0.970;2.963)	0.064
*Pg*	1.182(1.018;1.371)	**0.028**	1.107(0.908;1.349)	0.316
*Fn*	1.330(1.021;1.733)	**0.034**	1.068(0.753;1.515)	0.713
*Pi*	1.267(0.931;1.724)	0.132		
*Tf*	1.130(1.458;6.575)	**0.003**	2.570(1.140;5.794)	**0.023**
*Td*	1.331(0.998;1.721)	0.051		

Data are presented as odds ratio (OR) with corresponding 95% confidence interval (CI)

*Aa*, *Aggregatibacter actinomycetemcomitans*; aOR, adjusted odds ratio; *Pg*, *Porphyromonas gingivalis*; *Pi*, *Prevotella intermedia*; PPD, probing pocket depth; *Fn*, *Fusobacterium nucleatum*; *Td*, *Treponema denticola*; TE, treatment endpoint; *Tf*, *Tannerella forsythia*

Bold indicates statistically significant values (*P* < 0.05)

**Fig. 2:**
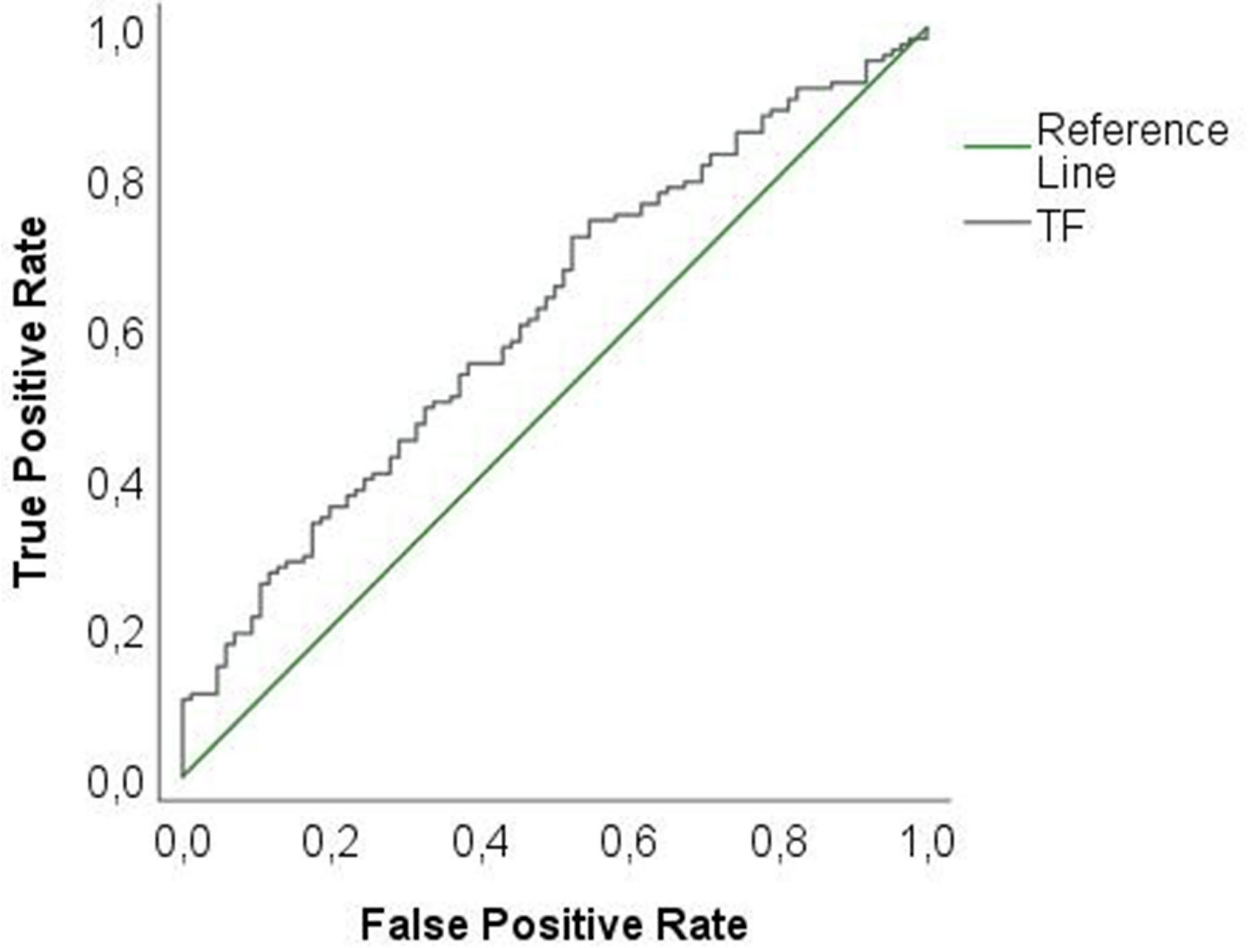
Receiver operating curve (ROC) analysis for successful and unsuccessful periodontal therapy using *Tannerella forsythia* (*Tf*)

For *Tf* a ROC analysis for successful and unsuccessful periodontal therapy showed an AUROC value of 0.615. According to the Youden index, the microbial load for *Tf* of 0.14 was the threshold level that provides the highest accuracy (Fig. 2).

## Discussion

### Key findings and objective

We aim to analyse the association between the baseline microbial load of selected periodontopathogenic bacteria and the outcome of first line therapy. The present study indicates a possible association between the mass of distinct periodontal pathogens and the therapy outcome after steps I and II of periodontal treatment. The findings suggest that specifically baseline *Tf* levels were associated with poorer treatment outcomes even after adjustment for factors known to compromise therapy. Taking this into account, patients with poorer response to therapy might be more reliably identified already at baseline.

### Discussion of methods and results

Regarding periodontal disease, a dysbiotic subgingival microbiome is mandatory for the manifestation and progression of periodontitis [35]. For determination of pathogenic subgingival infection various methods are available, i.e.culture-based methods [36], or sequencing of 16 S ribosomal RNA [17, 37, 38]. Herein, analysis of pathogenic subgingival bacteria was done by combining DNA amplification with a hybridization-technique, thus allowing a semi quantitative analysis [39, 40]. Due to the considerable intraindividual differences in the microbial composition of periodontal pockets [41], the analyses were done pooled per patient. This allows conclusions to be drawn at the patient level.

In this study, subgingival levels of pathogenic bacteria were associated with the achievement of the TE according to Feres et al. [31]. Using this definition of treatment success allows more easier comparison of the treatment response at a patient level and will enable future comparisons of results. Additionally, the endpoint defined by Feres et al. can be considered as “treat-to-target” endpoint and therefore resembles more closely a realistic endpoint after steps I and II therapy from a clinical point of view [30, 31, 42]. Almost 61%, of patients in this cohort, however, were unable to accomplish the selected TE. This appears as a poor overall success, but previously, an achievement of approximately 50% was reported as ideal and realistic [31]. Partially in line with the present findings, Benz et al. recently reported a success rate of 27% among patients not receiving systemic antibiotics as an adjunctive to non-surgical therapy using this endpoint [43]. A study by Bertel et al. used an even more rigid therapeutic endpoint defined by Chaple et al. (≤ 4 mm (no site ≥ 4 mm with BOP) and BOP < 10%) [30], observing that only 21% of patients achieved stability after active periodontal treatment. Moreover, after an observation period of 10 years, only 17% of patients remained stable according to the definition mentioned above [44]. Furthermore, the authors proposed entirely stable periodontitis after non-surgical therapy not to be achievable among patients with stages III and IV, which is in line with our results showing that only 0.9% of all patients presented without any periodontal pocket at re-evaluation [44].

Among patients who did not reach TE after steps I and II therapy, 26.5% were smokers, meaning that 66.7% of smokers were not successfully treated accordingly, confirming that smoking causes poorer results of periodontal treatment [12, 13].

The current data clearly show that patients who were unable to achieve TE after steps I and II therapy had a higher baseline burden of pathogenic bacteria. More detailed analysis revealed that four out of six bacterial species might increase the risk for treatment failure. However, after adjustment for various confounding variables only elevated levels of *Tf* remained independently associated with a > 2.5-fold increased risk for treatment failure. These findings are consistent with previous reports that have also observed less successful treatment outcomes associated with *Tf* [45, 46]. A reason for this could be the virulence profile of *Tf*, which in interaction with other bacteria could lead to less favourable healing [45, 47]. Overall, it appears that *Tf* in particular increases its pathogenic effect in the interaction of the whole biofilm [47].

The achievement of the therapeutic endpoint as proposed by Feres et al. is at least partially dependent upon the relative frequency of BOP [31]. Some pathogenic bacteria as considered herein have been previously associated with an increased prevalence of BOP [40] which is not confirmed by the current data not showing a linkage between the individual load of selected bacteria at baseline and the prevalence of BOP at REV, as categorized by Feres et al. (< 10%, 10–20%, and > 20% BOP) [31]. In line with the current results, a recently published meta-analysis concluded that the amount of bacteria, rather than the presence or absence of specific bacteria, was associated with the treatment outcome [16].

Taken together the current results appear to corroborate the concept of a critical biomass, sufficient to induce and maintain an immune response within the periodontal pocket ultimately leading to irreversible tissue destruction [16, 32, 48]. Loe et al. showed already that the development of gingivitis is a response to an increased microbial mass with a reproducible microbial succession leading to an increase in the proportional number of gram-negative microorganisms [49]. Similar to gingivitis, also periodontitis has been shown to correlate with specific shifts in the composition of the subgingival microbiome. Intriguingly, animal studies using a ligation-induced periodontitis model in mice have shown that increased microbial load is essential for triggering disease-associated T-helper immune responses that precipitate bone resorption, but that the increased microbial load alone is not sufficient to cause disease if it is not accompanied by specific changes in the overall structure of the microbial community [39, 40, 50]. Considering the interindividual differences in subgingival dysbiosis leading to periodontitis, the critical level for biomass might be different among patients [1, 23]. Moreover, the individual composition of the subgingival microbiome is subject to dynamic changes primarily caused by extrinsic influences, i.e. smoking, and oral hygiene measures.

Steps I and II therapy have the potential to reduce the level of bacteria under a critical threshold leading to temporary or final resolution of periodontitis-associated inflammation, ultimately leading to PC indicating treatment success [48, 51]. Accordingly, the critical level inducing disease recurrence might change during the course of therapy, specifically supportive periodontal treatment [1, 52]. Defining threshold values remains challenging due to variations in laboratory methods. A promising approach in this regard might provide the Subgingival Microbial Dysbiosis Index proposed by Chen et al. [53]. Furthermore, it was demonstrated that several pathogens are closely linked to the irreversible mediators of periodontal tissue destruction, which are frequently derived from neutrophils [54, 55]. Consequently, it would be intriguing to combine the microbial mass of these with, for instance, the active matrix metalloproteinase-8 point-of-care test to develop a potentially more effective biomarker-based index that can indicate therapeutic interventions [55, 56].

### Limitations

The present study has several limitations that might specifically impair the generalisability, applicability and transferability of the present results. As a monocentric observational study, the generalisability of the findings is limited. Furthermore, due to incomplete data collection, periodontal grading was not applied on diabetic patients. In addition, data were collected and patients were treated between 2013 and 2016. Therefore, the only data that could be reported were those included in the primary protocol. As a result, active matrix metalloproteinase-8 point-of-care test results are not available for these patients. In the present cohort, periodontal treatment was carried out in an undergraduate program under the supervision of experienced periodontists, which might affect the comparability of the data. Considering the therapeutic outcomes commonly achieved by general dentists or hygienists, with varying levels of skills and experience, the overall therapy success as found in the current study cohort are deemed satisfactory [7, 57]. Additionally, the microbial load seems to increase with the severity of periodontal disease and patients with more severe periodontitis are at higher risk for treatment failure [7, 39]. To mitigate this bias, this study considered stage III periodontitis only. However, it is important to take into account that this bias might still be relevant since patients with different individual levels of severity within stage III periodontitis have been included into [11].

## Conclusion

The rationale for the use of microbial biomarkers in periodontal diagnosis has been discussed controversially in the literature. The results of the present study indicate that besides the probing depth at baseline, an increased microbial load is associated with a higher risk for treatment failure after steps I and II therapy. Within the limitations of this study, specifically, baseline *Tf* levels were associated with poorer treatment outcomes and might thus improve the accuracy of periodontal diagnosis. However, further research is needed to define an individual level of critical biomass in each patient, that can potentially work as an indicator for periodontitis before manifestation.

## Data Availability

No datasets were generated or analysed during the current study.The data that support the findings of this study are available from the corresponding author upon reasonable request.
